# Aggregation of experts: an application in the field of “interactomics” (detection of interactions on the basis of genomic data)

**DOI:** 10.1186/s12859-018-2447-0

**Published:** 2018-11-21

**Authors:** Sinan Abo Alchamlat, Frédéric Farnir

**Affiliations:** Department of Biostatistics, Faculty of Veterinary Medicine, University of Liège, Sart Tilman B43, 4000 Liege, Belgium

**Keywords:** Gene-gene interaction, Epistasis, Single nucleotide polymorphism, Genome-wide association study, Multi dimensional reduction, K-nearest neighbors

## Abstract

**Background:**

Despite the successful mapping of genes involved in the determinism of numerous traits, a large part of the genetic variation remains unexplained. A possible explanation is that the simple models used in many studies might not properly fit the actual underlying situations. Consequently, various methods have attempted to deal with the simultaneous mapping of genomic regions, assuming that these regions might interact, leading to a complex determinism for various traits. Despite some successes, no gold standard methodology has emerged. Actually, combining several interaction mapping methods might be a better strategy, leading to positive results over a larger set of situations. Our work is a step in that direction.

**Results:**

We first have demonstrated why aggregating results from several distinct methods might increase the statistical power while controlling the type I error. We have illustrated the approach using 6 existing methods (namely: MDR, Boost, BHIT, KNN-MDR, MegaSNPHunter and AntEpiSeeker) on simulated and real data sets. We have used a very simple aggregation strategy: a majority vote across the best loci combinations identified by the individual methods. In order to assess the performances of our aggregation approach in problems where most individual methods tend to fail, we have simulated difficult situations where no marginal effects of individual genes exist and where genetic heterogeneity is present. we have also demonstrated the use of the strategy on real data, using a WTCCC dataset on rheumatoid arthritis.

Since we have been using simplistic assumptions to infer the expected power of the aggregation method, the actual power we estimated from our simulations has turned out to be a bit smaller than theoretically expected. Results nevertheless have shown that grouping the results of several methods is advantageous in terms of power, accuracy and type I error control. Furthermore, as more methods should become available in the future, using a grouping strategy will become more advantageous since adding more methods seems to improve the performances of the aggregated method.

**Conclusions:**

The aggregation of methods as a tool to detect genetic interactions is a potentially useful addition to the arsenal used in complex traits analyses.

**Electronic supplementary material:**

The online version of this article (10.1186/s12859-018-2447-0) contains supplementary material, which is available to authorized users.

## Background

Major technical advances have made genetic information from molecular origin easily available to the research community in the last decades. In this new context, where very large datasets from the lab are available, the challenge is progressively shifting from data acquisition to data management and use. Genetic mapping - the association of genetic polymorphisms to phenotypic variations - is one of the major goals targeted by geneticists, and strongly benefits from this recent data explosion. Despite remarkable successes - such as the discoveries of mutations involved in breast cancers, for example - we still need new approaches and new strategies to deal with situations that are more complex. These complex situations include those where several genes interact, making the relationship between the genomic pattern and the corresponding phenotypic variations not easy to identify. Although researchers have proposed many methods to tackle this difficult problem, and despite some successes, no gold standard method is currently available: some methods might be efficient while other fail in a set of situations, but the reverse might be true in another set of situations. Consequently, combining the performances of various methods seems an appealing approach. Since a large portion of the genetic determinism underlying many traits of interest in various organisms, including humans, is still unknown and uncharacterized, genetic mapping and positional cloning is a very active field of research [1]. A classical approach in this field is the use of genome-wide association studies (GWAS): dense molecular markers maps (most often, large sets of Single Nucleotide Polymorphisms (SNP), but not exclusively) are used to scan the whole genome and associations of markers with the trait of interest are sought. Although successful in many studies [2], this approach has not been successful in many other cases, even when complete genomic information (i.e. sequence data) was available. Several reasons might explain this situation, such as a small power to detect effects of modest size or oversimplified statistical models [3]. If increasing the cohorts sizes used for mapping is difficult or useless, a possible track to tackle this “missing heritability” problem might be to fit more elaborate models, such as those introducing epistatic or gene-environment interactions [4, 5]. Genes interactions are interplays between two or more genes with an impact on the expression of an organism’s phenotype. They are thought to be particularly important to discover the genetic architecture underlying some genetic diseases [4, 5]. Consequently, there has been an increased interest in discovering combinations of markers that are strongly associated with a phenotype even when each individual marker has little or even no effect [6]. This approach faces at least two problems: first, modeling and identifying every (or even any) interaction is a potentially very challenging task in today situations where very large sets of markers (up to several millions) might be available. Note that large sets of markers are usually necessary in association studies for a complete characterization of the tested genomic regions. Second, from a more statistical point of view, fully modeling the complexity leads to models with a large dimensionality, leading to the well-known ‘curse of dimensionality’ problem [7]: in rough words, the accurate estimation of an increased number of parameters is hampered by the reduced sizes of the tested cohorts. Many methods (such as multifactor dimensionality reduction approach using K-Nearest Neighbors (KNN-MDR) [7], multifactor dimensionality reduction (MDR) [8], MegaSNPHunter [9], AntEpiSeeker [10], BOolean Operation-based Screening and Testing (BOOST) [11], Bayesian epistasis association mapping (BEAM) [12], BHIT [13], Random forest (RF) [14], among others) have nevertheless been proposed for detecting such interactions. Despite successes of these methods to unravel some genetic interactions [3], no unique method has emerged to detect most of the interactions so far. Furthermore, the relative performances of these methods remain largely unclear and necessitate more investigations. As a step in that direction, we propose using a method based on the principle of the aggregation of experts, where the “experts” would be a set of popular published methods. In parallel, we highlight some of the features of the individual methods and discuss possible aggregation strategies.

## Methods

Methods of aggregation are not new and have been used extensively to improve classification [15, 16]. They are a popular research topic in supervised learning and useful for constructing good ensembles of classifiers [17]. In our study, we have used aggregation to combine the results of various popular gene-gene interactions mapping methods and assessed the performances of this approach. The idea of the method is to combine the information from a few methods in order to create new consensual knowledge [18]. The aggregation of experts, which is an instance of the larger class of ensemble methods where aggregation is the technique allowing to combine information from multiple sources, has been shown to yield more accurate and robust predictions than individual experts on a variety of classification problems [19]. Using this approach, it is often possible to decrease the amount of redundant data, to filter out wrong results (false positives and false negatives) and to increase the accuracy of the results [20]. In this paper, we investigate the aggregation of published gene interactions mapping methods (described below). As can be found in the literature, available methods have pros and cons, and no unique method is uniformly better than the others to detect genetic interactions. Our objective was therefore to obtain a comprehensive method able to detect more true positive interactions than each individual method by combining the strengths of these individual approaches while better avoiding false positive results. The very simple idea is therefore to let each method run independently and finally propose a final decision based on some consensus obtained from the individual methods results. An easy example of such a consensus is the use of the most frequent opinion as the aggregated expert’s opinion. We have used this approach in our experiments.

The major objective of the aggregation strategy is thus to obtain higher detection power than the individual methods used in the aggregation while conserving an acceptable type I error. In other words, we want to increase both the sensitivity and the specificity of the method when compared to individual approaches. We can obtain, using some assumptions, a rough estimate of an upper bound for the power as follows. Assume runs are performed on Q (≥ 2) methods, where each method has a power p_i_, *i* = 1, ..., Q. If we assume that the methods are independent (the results obtained using one method gives no indication on what can be expected from another one; this assumption is discussed below):

- the probabilities p_i_ can be multiplied to model situations where two or more methods correctly identify a combination associated to the phenotype,
- it is unlikely that 2 or more independent methods would identify the same false positive combination, given that the number of potential combinations is huge in most practical situations.

Using the second assumption, we will then consider that an interaction is detected as soon as at least 2 of the Q methods detect the same combination. Next, if we consider that 2 results are possible for each method (correct identification of a causative combination = 1, incorrect identification of the causative combination = 0), 2^Q^ situations are possible for the aggregated expert: (0, 0, ..., 0), (1, 0, ..., 0), ..., (1, 1, ..., 1). Each of these k situations (s_1_, s_2_, ..., s_Q_) has a probability ${P}_k=\prod \limits_{i=1}^{i=Q}{p}_i^{s_i}\ast {\left(1-{p}_i\right)}^{\left(1-{s}_i\right)}$ and the power of the aggregated method is obtained by summing these Pi over the set Ω of all situations where at least 2 methods are successful:

1 $$ P=1-{\prod}_{i=1}^{i=Q}\left(1-{p}_i\right)-{\sum}_{i=1}^{i=Q}\frac{p_i}{1-{p}_i}\ast {\prod}_{j=1}^{j=Q}\left(1-{p}_j\right)=1-{\prod}_{i=1}^{i=Q}\left(1-{p}_i\right)\ast \left(1+\sum \limits_{i=1}^{i=Q}\frac{p_i}{1-{p}_i}\right) $$

The Table 1 illustrates this result in (theoretical) situations where all the individual methods have the same power.

**Table 1:**
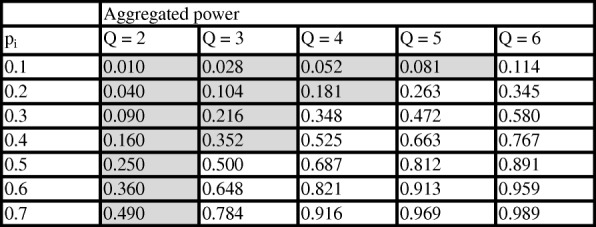
Aggregated power as a function of the individual methods power pi (assumed identical) and the number Q of methods

Highlighted cells correspond to situations where the aggregated method has lower power than the individual ones. Individual methods are assumed to be independent

In this table, the independence assumption penalizes the aggregated expert in situations where the number Q of methods is low and the individual powers are low (these situations correspond to the grayed cells). On the other hand, adding methods increases substantially the power, especially when the individual powers are high.

In most practical situations, methods will not be independent and the power gains will differ from the predictions of formula (1). We have performed some simulations to see the effect of the correlation between the methods results on the power of the aggregated method: results show that although the power decreases when the correlation increases, it remains in most cases above the power of individual methods (see Additional file 1). In summary, the performance of the aggregated method will depend on the individual methods performances, on the number of methods but also on the correlation between the methods results. These correlations can be assessed using simulations, either directly – by counting situations where methods provide concordant results above what is expected by chance only – or indirectly – by comparing the simulations results to what is expected under the hypothesis of independent methods (Table 1). A correlation measure could be based on Cohen’s kappa measure [21]:

$$ k\left(i,j\right)=\frac{\#\left(\dots, {1}_i,\dots, {1}_j,\dots \right)+\#\left(\dots, {0}_i,\dots, {0}_j,\dots \right)-N\ast {p}_i\ast {p}_j-N\ast \left(1-{p}_i\right)\ast \left(1-{p}_j\right)}{N-N\ast {p}_i\ast {p}_j-N\ast \left(1-{p}_i\right)\ast \left(1-{p}_j\right)} $$

where #(…, 1_*i*_, …, 1_*j*_, …) is the number of simulations where methods i and j simultaneously provide a positive result, #(…, 0_*i*_, …, 0_*j*_, …) is the number of simulations where methods i and j provide a non-positive result, N is the number of simulations, and *p*_*i*_ and *p*_*j*_ are the powers of methods i and j, respectively.

In order to cover a range of situations where aggregation could be useful (see Table 1), our work is based on six methods that have been published and used to detect interacting genetic loci involved in the genetic determinism of a trait. A short description of each of these methods is given below, and details can be found in the corresponding publications:

1. MDR: The Multi-Dimensional Reduction (MDR) method is designed to replace large dimension problems with reduced dimension ones, allowing to make inferences based on a smaller set of variables [8].
2. KNN-MDR is an approach combining K-Nearest Neighbors (KNN) and Multi Dimensional Reduction (MDR) for detecting gene-gene interactions as a possible alternative, especially when the number of involved determinants is high [7].
3. BOOST (Boolean Operation-based Screening and Testing), is a two-stage method (screening and testing) using Boolean coding to improve the computational performances [11].
4. MegaSNPHunter (MSH) uses a hierarchical learning approach to discover multi-SNP interactions [9].
5. AntEpiSeeker (AES) is an heuristic algorithm derived from the generic Ant Colony Optimization family [10].
6. BHIT uses a Bayesian model for the detection of high-order interactions among genetic variants in genome-wide association studies [13].

Although other methods, such as support vector machines (f.e. [22, 23]), neural networks (f.e. [24, 25]), decision trees (f.e. [26]), random forests (f.e. [27, 28]) among others, have been developed and could be used in the aggregation, we limited ourselves to the methods described above. A first reason for this choice is that using only 6 methods should allow to see the benefits from the aggregation strategy, as shown above, while limiting the computer load. Another reason for the choice of these 6 methods is that they mostly cover the panel of the available search strategies: parametric (BHIT) and non-parametric (the others), exhaustive searches (MDR and BOOST), stochastic search (MegaSNPHunter), heuristic approach (AntEpiSeeker). Furthermore, they have been applied successfully to real datasets and software is available for each of these methods.

Since we wanted to assess the performances of the aggregation method and compare them to the results of the individual methods, we have performed simulations. We now describe these simulations.

### Simulations

One of the aims of our study was to assess the performances of the methods to unravel gene-gene (or gene-environment) interactions in the absence of large marginal effects. The reason for that choice was that many methods are able to detect such large marginal effects and to infer interactions within a limited set of loci selected on that basis. Accordingly, we wanted to devise an approach that is able to detect interactions even in the absence of marginal effects. For that reason, efforts have been devoted to generate datasets with interacting genes in the absence of significant marginal effects. Note that this is not a restriction on the use of the aggregation strategy: the presence of marginal effects is likely to increase the power of the individual methods, and consequently to have a positive influence on the power of the aggregated method. We simply put ourselves in a difficult situation where improvements were needed. Furthermore, heterogeneity between samples has been shown to be a major source for the non-reproducibility of significant signals [29]. We have modeled heterogeneity by associating penetrances (i.e. Pen = probabilities of a phenotype given a genotype) to the multi-locus genotypes underlying the simulated binary trait. Consequently, individuals carrying the causal alleles could be affected (with a probability equal to Pen) or not.

**Fig. 1:**
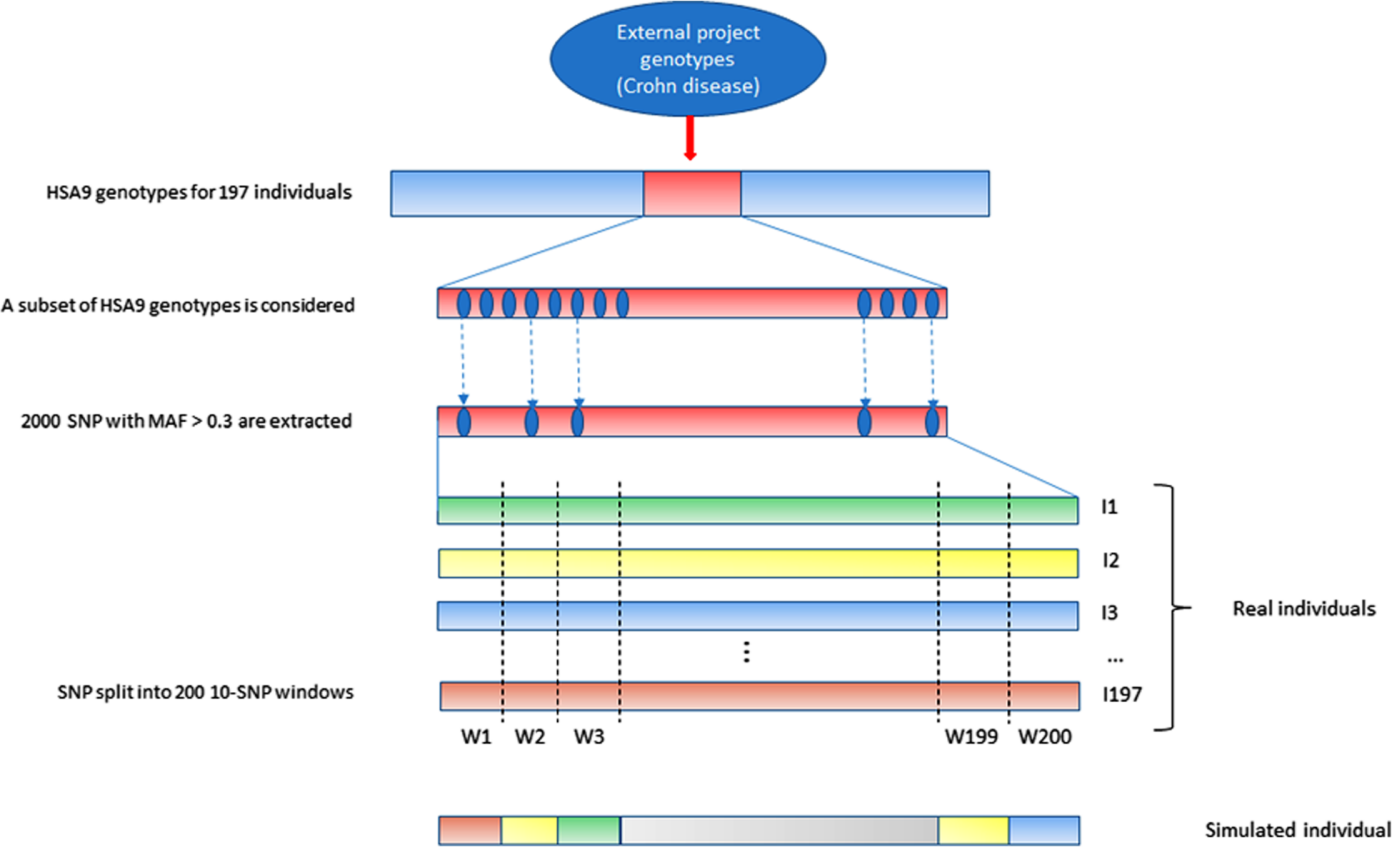
Genotypes generation using a real dataset. Simulated genotypes are a concatenation of 200 windows, containing 10 SNP each, obtained from real individuals genotypes

**Fig. 2:**
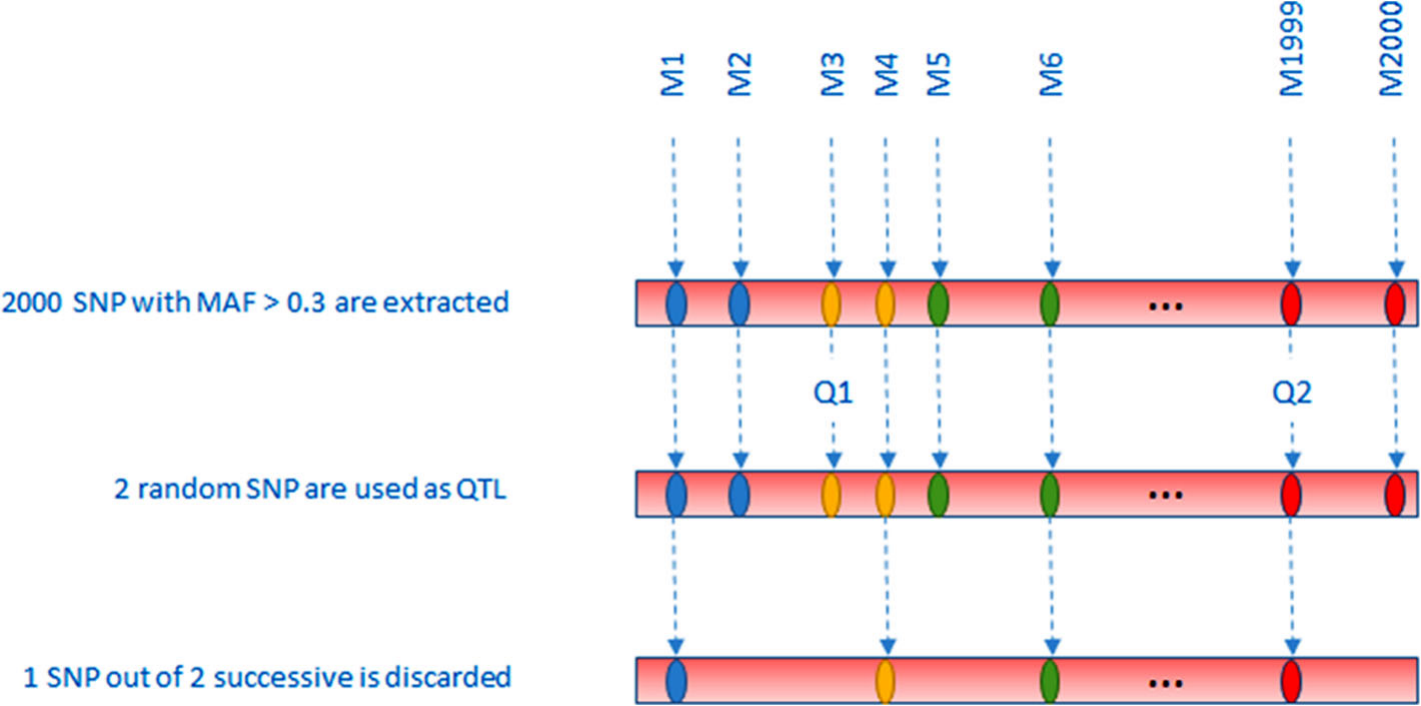
QTL (Q1, Q2) used as a basis to generate the interaction. In this example, QTL Q1 has been discarded, but QTL Q2 is still present in the final genotyping dataset. The phenotype is defined using the penetrance function corresponding to this 2 SNP

**Fig. 3:**
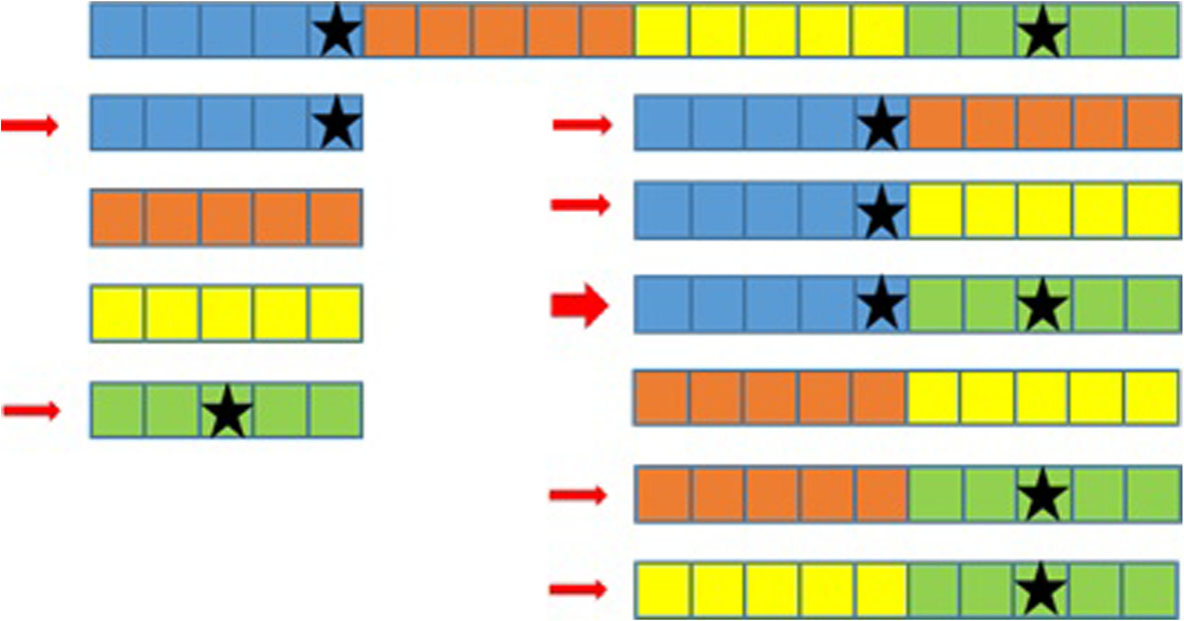
An example with 20 SNP (represented by squares) partitioned into 4 groups (represented by the colours) of 5 SNP. The causative SNP are marked with a star. All combinations of 1 or 2 groups are shown, those bearing a causative SNP are marked with a small arrow, and the optimal with the 2 causative SNP with a big arrow

The process can be split into 4 steps:

1. Genotypes generation (see Fig. 1).
  1. Genotyping data from a study on Crohn disease in Caucasians [30] has been obtained for 197 individuals.
  2. SNPs spanning a genomic region on chromosome 9 (HSA9) have been extracted, and, to decrease the computational requirements of the simulations, a subset of 2000 informative markers has been selected for our simulations. In order to recover a large part of the information lost in subselecting markers, only markers with a MAF > 0.3 and no missing genotypes have been selected. Subsequent tests (Hardy-Weinberg equilibrium, recovery of a significant linkage disequilibrium) have been carried on to validate the finally used subset (data not shown).
  3. Since many different individuals are needed in the simulations, we have used a trick similar to [6] to generate new individuals based on the few (i.e. 197) available genotypes: each individual genotype was chopped into 10 SNP windows, leading to 200 windows. Consequently, each window has (maximum) 197 different 10-loci genotypes. We then built each simulated individual genotype by randomly sampling one of the 197 possible 10-loci genotype for each of the 200 windows and concatenating the 200 10-loci genotypes into a new complete genotype with 2000 markers. This technique allows for 197^200^ potentially different individuals while conserving some LD.
2. Phenotypes generation (see Fig. 2).
  (4) 2 SNP were randomly chosen as having an effect on the simulated phenotype (although not a limitation of the method, we restricted our study to 2-SNP interactions). Note that, since SNP selection was random, SNP could be linked or not.
  (5) Selected SNP genotypes were used to generate the binary phenotypes. The details of the algorithm are given in [7], but, in summary, after generating 2-locus penetrances (Pen) leading to approximately no marginal effect, a uniformly distributed random number R is sampled between 0 and 1 and compared to the penetrance Pen of the simulated 2-locus genotype: if R < Pen, the simulated individual is supposed to be a case (1). If not, it is a control (0)
  (6) One SNP out of 2 consecutive SNPs was then randomly discarded, leaving 1000 markers genotypes for the analyses. The rationale of this selection is that causative mutations might nowadays be present or not in the genotyped variants. This will also be the case in our simulations (see Fig. 2).
3. Statistics computation and significance assessment.
  (7) The genotypes and corresponding phenotypes were then studied using all 6 methods.
    - KNN-MDR splits the 1000 SNP into 100 windows of 10 consecutive markers and measures the association between each combination of 1 (100 tests) or 2 (4950 tests) windows with the phenotype using balanced accuracy [7]. Among all possible combinations, the one considered as optimal is the one containing both causative SNP (see Fig. 3).
    - The other approaches use their own statistics to rank the tested combinations associations with the phenotype from strongest to weakest (see [8–11, 13] for details).
  (8) We assessed significance using 100 permutations of the phenotypes for each simulation. Permutation of the phenotypes with respect to the genotypes breaks the potential relationship between phenotypes and genotypes. Accordingly, analyses on permuted data correspond to analyses under the null hypothesis of no association. We kept the highest value of the statistic obtained in each permutation to build the distribution under the null hypothesis, and then compared the statistics obtained with the real (i. e. non permuted) data to this distribution to obtain a *p*-value for the tested combinations. Although this number of permutations is too low for routine work, it was used to reduce the computing burden and help us to discriminate between results clearly non-significant (i.e. *p* > 0.05) and those potentially significant (i.e. *p* < 0.05). When a higher precision was needed for the *p*-values (see below for real data), an adaptative permutations scheme was used, in which windows not reaching a pre-determined *p*-value threshold are progressively abandoned in the permutations scheme since these windows are very unlikely to finally reach a significant result [31].
4. Aggregation of the results.
  (9) After completing the simulation and the permutations for each method, we performed a majority vote among the obtained optimal combinations. If one combination obtained the majority, it became the aggregated method’s chosen combination. When no majority could be obtained, the aggregated method failed to obtain a solution (see simulation in Table 2 as an example).

**Table 2:**
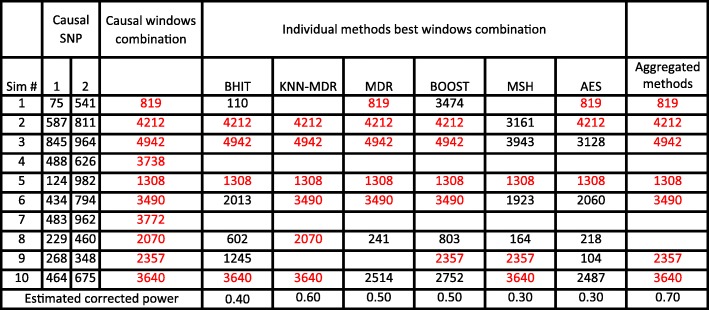
A sketch of the results from ten simulations

Causative SNP are given by their location in the list (between 1 and 1000). Detected combinations are shown as a number representing one of the 5050 combinations. Empty cells correspond to the situations where no significant combination was found. The correct solutions are written in red and the ‘-‘correspond to failures of the aggregation (no majority)

Note that the combinations mentioned in this section correspond to KNN-MDR windows combinations. Since the 5 other methods report combinations of SNP, these combinations of SNP are mapped to the corresponding combinations of windows before performing the majority votes.

In each simulation, we generated genotypes and phenotypes to obtain 500 cases and 500 controls and analyzed the simulated data using the approach described above.

This whole process was repeated 1000 times in order to obtain an accurate estimator of the corrected power, where the “corrected power” is estimated as the proportion of situations where the methods (including the “aggregated expert”) correctly identify the causative combination. Table 2 illustrates the decision scheme using the 6 individual methods and the aggregation method on the few first simulations.

### Real data

Analyses using real data have been performed on a Rheumatoid arthritis (RA) genotype dataset involving 1999 cases and 1504 controls obtained from WTCCC [32]. Genotypes from the Affymetrix GeneChip 500 K Mapping Array Set have been filtered using the usual quality controls tests on DNA quality (percentage of genotyped marker for any given individual above 90%), markers quality (percentage of genotyped individuals for any given marker above 90%), genotypes frequencies (markers with a *p*-value below a Bonferroni adjusted 5% threshold under the hypothesis of Hardy-Weinberg equilibrium in the controls cohort have been discarded). Missing genotypes for the GeneChip markers have been imputed using impute2 software [33]. This procedure led to 312,583 SNP to be analyzed for the 2 cohorts. Working with such a large panel remains quite challenging for several of the methods we have been using in this study. Therefore, we decided to reduce the number of SNP to about 52,000 by roughly considering the SNP with the highest MAF in each window of 6 successive SNP. Of course, in future studies, when more performant methods will be available (such as KNN-MDR, among others), the complete set of SNP could be considered again. Alternatively, after targeting some regions with this reduced set of SNP, the discarded SNP could be reintroduced in order to refine the location of the regions of interest.

Next, we used each method described above on this dataset as follows:

- MDR tested all combinations of 2 SNP (i.e. more than 1,350,000,000 combinations) and sorted the results by decreasing balanced accuracies. To obtain significance, we used a Bonferroni correction as is done in the MDR package: we kept the first 5000 highest balanced accuracy results, and used the corresponding 5000 combinations to perform the permutations. The number of permutations was conservatively based on the total number of tests, leading to a corrected *p*-value equal to 3.698225× 10^− 11^. This necessitated to perform 10^11^ permutations.
- KNN-MDR has been used first on 1000-SNP windows, leading to 1326 tests involving 2 windows. Using an adaptative permutations scheme as is done in [7], and progressively decreasing the windows sizes, we ended up with a set of 33 windows containing 50 SNP each. Finally, a MDR approach was performed involving all combinations of 2 SNP from this set of 1650 SNP (i.e. 1,360,425 combinations).
- MegaSNPHunter has been used with the same parameters and using the same approach as has been done in a previous GWAS study [9], and the results have been sorted by decreasing χ^2^ values. To obtain significance, we performed a Bonferroni correction for the first 5000 results, similarly to what has been done for MDR.
- AntEpiSeeker has also been used with the same parameters and using the same approach as been done in a previous GWAS study [10], and the 5000 larger χ^2^ were kept to perform the simulations as done in MDR.
- BOOST has also been used with the same parameters and using the same approach as as been done in a previous GWAS study [11], with the results sorted by decreasing values of Kirkwood superposition approximation (KSA). To obtain significance, we performed a Bonferroni correction for the first 5000 results, and then used the same permutations approach as for the other methods.

## Results

### Results on simulated data

#### Power

**Fig. 4:**
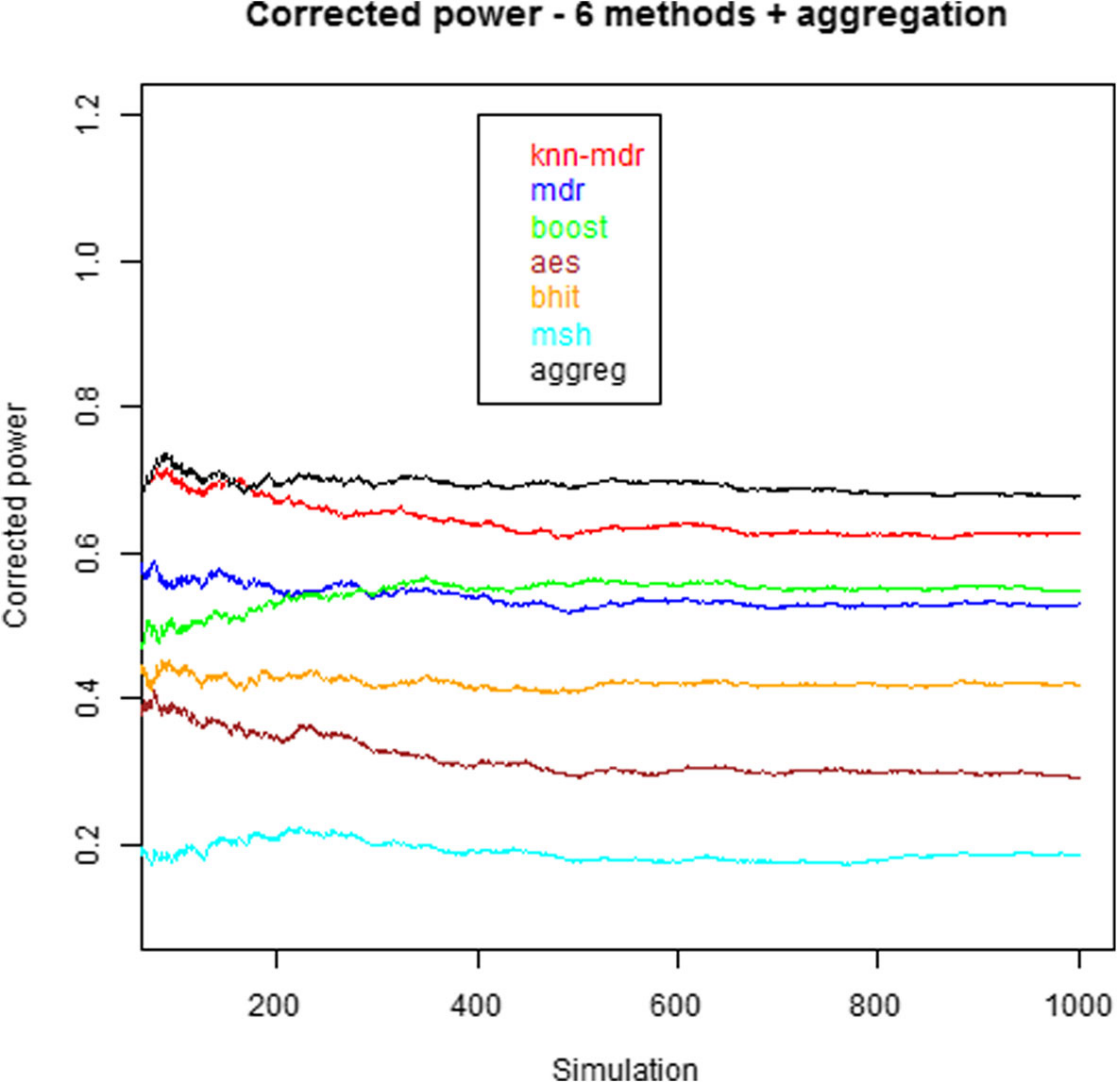
Estimations of the corrected power for the 6 individual methods and the aggregation method. The 100 first simulations lack estimators stability and are not shown. Final powers are 0.628, 0.530, 0.549, 0.293, 0.419, 0.186 and 0.678 for KNN, MDR, Boost, AntEpiSeeker, BHIT, MegaSNPHunter and the aggregation method, respectively

**Fig. 5:**
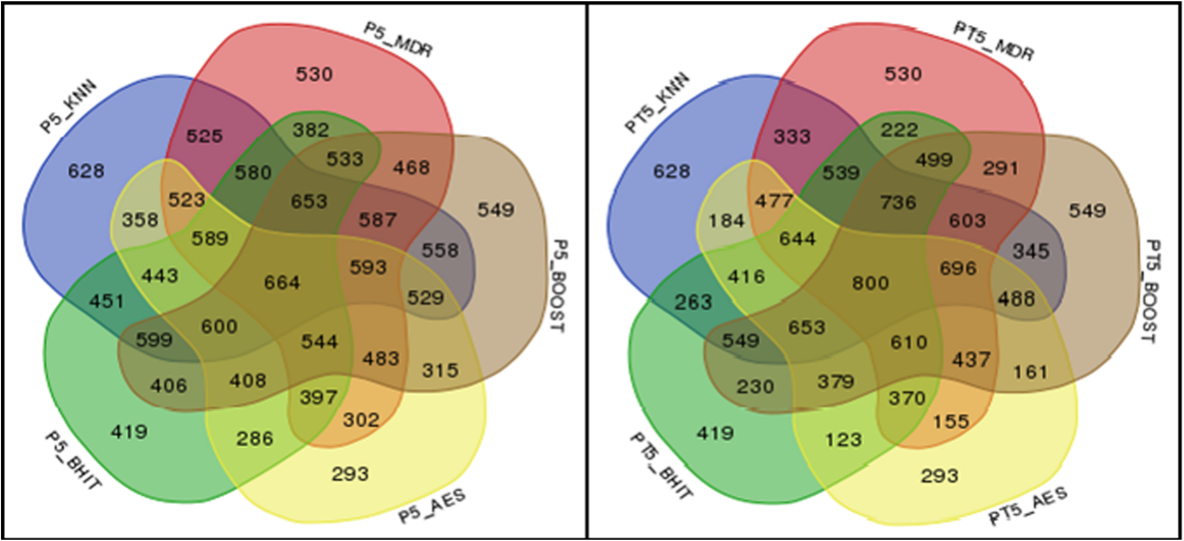
Power (in ‰) of 5 individual methods (KNN, MDR, BOOST, AntEpiSeeker, BHIT) and of the 26 possible combinations of aggregated methods. The left diagram shows the results obtained in the simulations, while the right diagram shows the expected results under the hypothesis of methods independence. Note that the latter does not necessarily correspond to a majority vote

Figure 4 shows the estimations of the corrected power as a function of the number of simulations. After a few hundreds simulations, the estimations stabilize and the relative ranking of the methods in terms of corrected power becomes fixed. The aggregation method is more powerful than any of the 6 other methods in our simulations. Another more detailed representation of the results is provided in Fig. 5. Since the representation of more than 5 simultaneous methods is difficult and of no visual help, we have omitted the results involving MegaSNPHunter in the figure (the results with the 6 methods are provided in the Additional file 2).

In the setting used to obtain the Fig. 5 results (i.e. using 5 individual methods), the highest empirical power (0.664) is obtained for the aggregation expert involving the 5 methods. The power of the individual methods used in the theoretical predictions obtained using (1) are the empirical powers of these methods, explaining why these are equivalent in the two graphs. It can also be observed that all powers of the aggregated methods involving only two methods are higher than expected. When three methods are involved, the powers are sometimes higher, sometimes lower than expected under independence. For four or five methods, the powers are constantly lower than expected, although higher than for any individual method when the five individual methods are aggregated (and even higher for six methods, 0.678, as mentioned on Fig. 4).

**Fig. 6:**
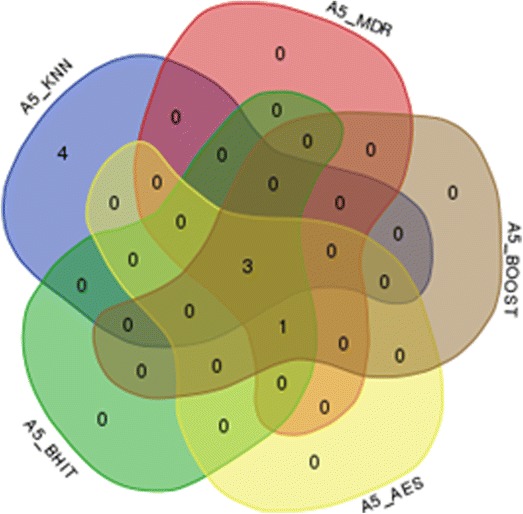
Specific positive results for each (combination of) method(s) in 1000 simulations. The numbers denote the number of simulations where the corresponding (set of) method(s) was the only one to indicate the correct combination

Figure 6 shows the number of simulations (within the 1000 simulations) where only one method or combination of methods discovered the proper combination.

#### False positive rates

A second incentive for using aggregation is that false positive rates are likely to decline due to the use of a majority vote among parallel results: false positive results obtained using one method might disappear when using a different method, with a different rationale. In our work, we have assessed two different kinds of false positive results:

- Either the methods identified an incorrect combination (note that these incorrect results are not included in the previous results on “corrected” power), generating an incorrect positive result.
- Either they identified a combination when no effect had been simulated (i.e. found a “false positive” result).

**Fig. 7:**
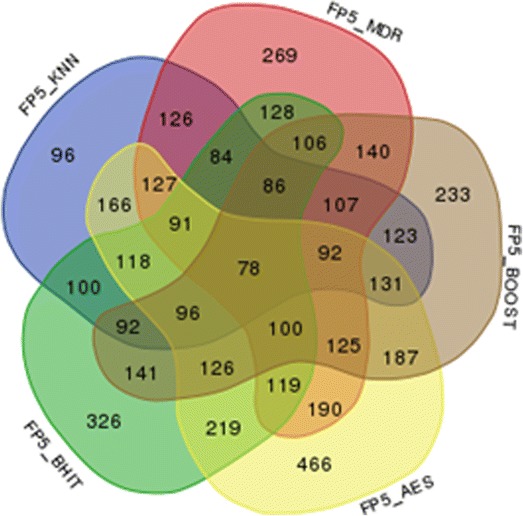
Number of incorrect positive results in 1000 simulations at the 5% threshold. A result is an incorrect positive result when the most significant combination (if any) does not correspond to the simulated combination. The number of incorrect positive results falls to 47 when MegaSNPHunter results are added

To test the first type of incorrect results, we have used the same set of simulations as for the power results and counted the number of false positives for each scenario. The combination identified as the most significant, if any, was taken as the solution for each of the methods, and the one with a majority vote, if any, for the aggregated method. The results are reported in Fig. 7.

**Fig. 8:**
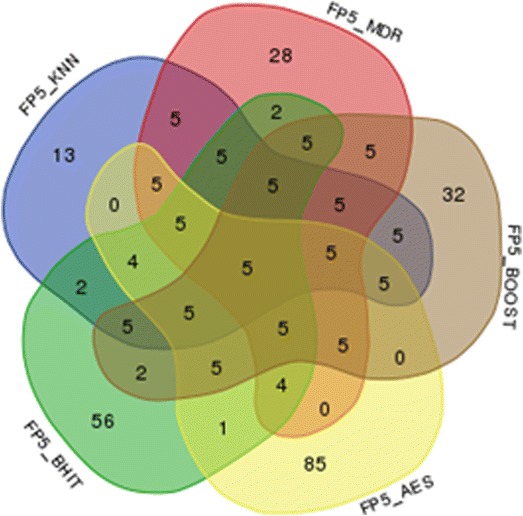
Number of simulations providing false positive results (significance threshold = 5%) in a set of 200 simulations. An approximate 95% confidence interval for the number N of false positive results is [4; 15]

For the second definition, we have simulated 200 situations where no SNP was involved to generate the phenotype. Results are reported in Fig. 8.

**Fig. 9:**
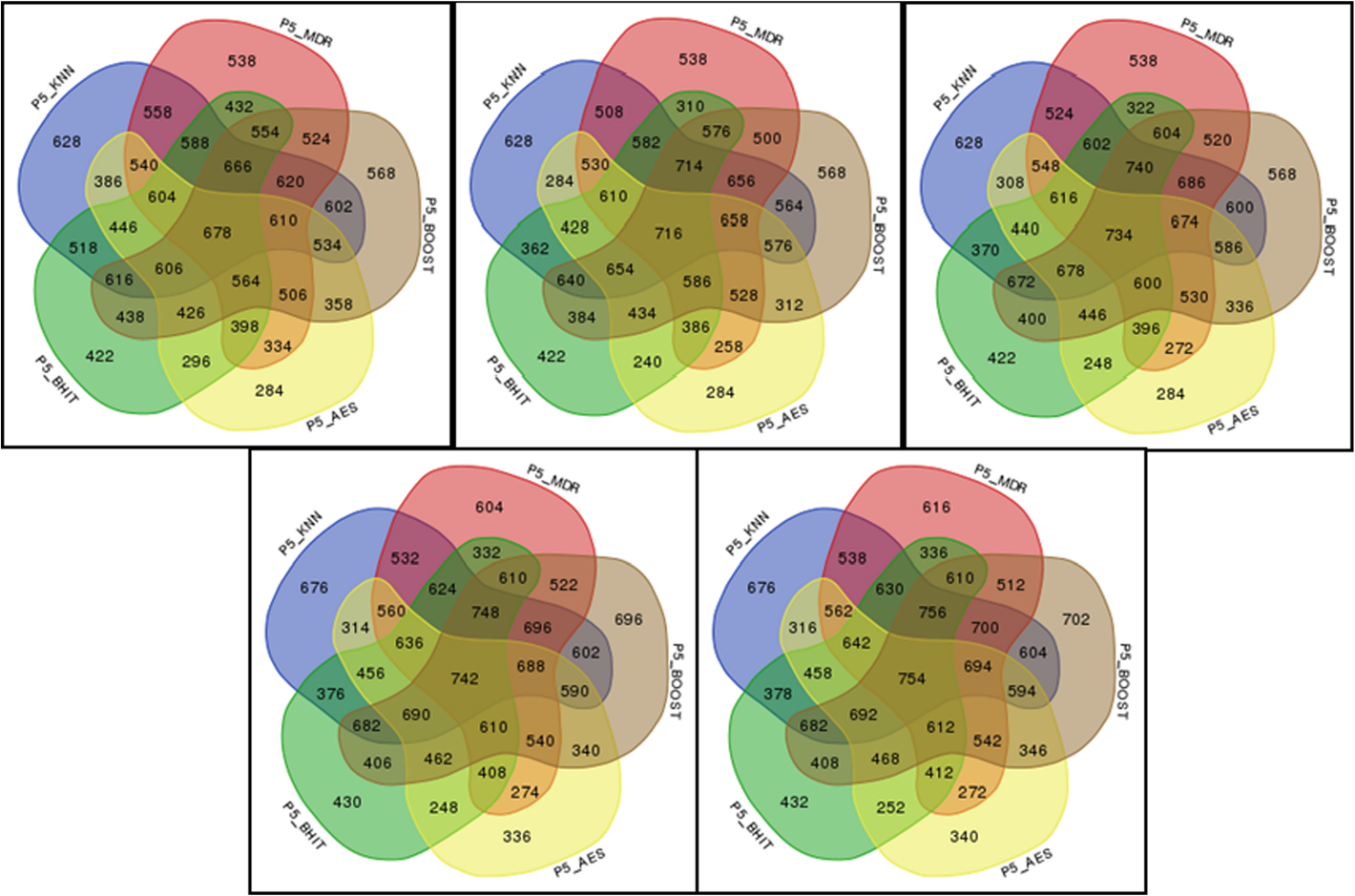
Powers (in ‰) of 5 individual methods (KNN, MDR, BOOST, AntEpiSeeker, BHIT) and of the 26 possible combinations of aggregated methods when the number of kept significant combinations varies from 1 (top left) to 5 (bottom right)

**Fig. 10:**
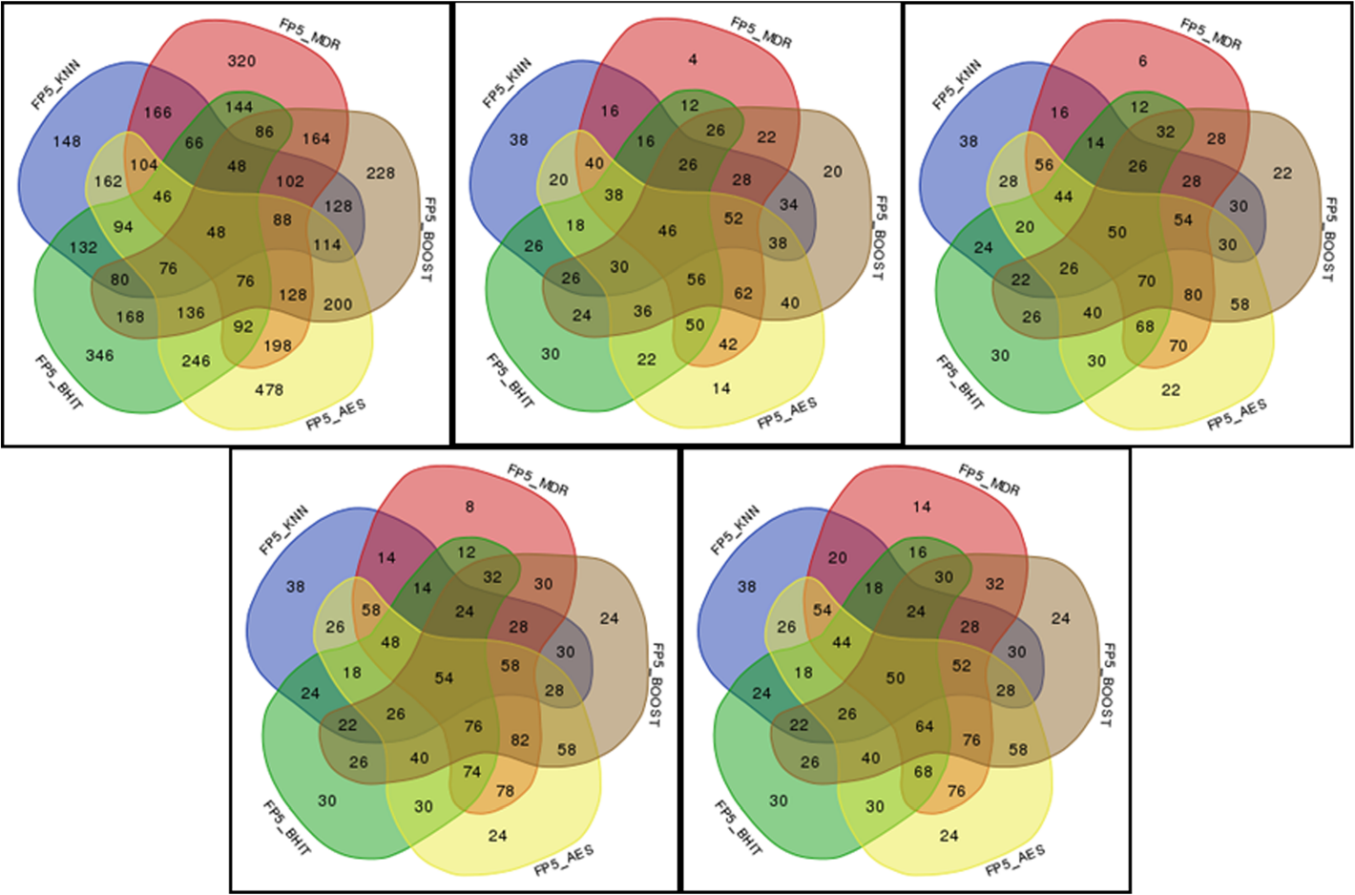
False positive rates (in ‰) of 5 individual methods (KNN, MDR, BOOST, AntEpiSeeker, BHIT) and of the 26 possible combinations of aggregated methods when the number of kept significant combinations varies from 1 (top left) to 5 (bottom right)

We carried out a second set of 500 simulations. In these analyses, we kept up to the 5 most significant combinations to see whether checking more than “the best” combination allows improving the (corrected) power without harming too much the false positives rate. Figures 9 and 10 present the results of these simulations.

#### Correlation

Correlations between the methods results have been computed using the Cohen’s Kappa approach described above. The results are presented in Table 3. The correlations have been computed for each combination of 2 methods, and for 1 to 5 kept top-ranked markers combinations. We have assessed the significance of these measures by permuting 1000 times the results (success or failure) for each method and computing the corresponding values of kappa. For all combinations of methods and sets of markers combinations, no permuted kappa reached the value obtained with the real data, indicating that all *p*-values are lower than 0.001. Consequently, even when the methods show a slight agreement (κ < 0.200), the methods are very significantly correlated.

**Table 3:** Cohen kappa coefficients for all combinations of methods using the approach given above

Cohen k	1 region	2 regions	3 regions	4 regions	5 regions
KNN-MDR	0.587	0.537	0.518	0.515	0.511
KNN-BOOST	0.485	0.459	0.502	0.493	0.486
KNN-AES	0.244	0.230	0.246	0.247	0.245
KNN-BHIT	0.177	0.186	0.192	0.200	0.203
KNN-MSH	0.179	0.159	0.152	0.147	0.156
MDR-BOOST	0.608	0.512	0.497	0.507	0.469
MDR-AES	0.354	0.357	0.353	0.349	0.345
MDR-BHIT	0.238	0.255	0.263	0.270	0.275
MDR-MSH	0.208	0.174	0.156	0.155	0.170
BOOST-AES	0.343	0.287	0.277	0.287	0.283
BOOST-BHIT	0.319	0.308	0.287	0.298	0.299
BOOST-MSH	0.195	0.166	0.161	0.158	0.162
AES-BHIT	0.443	0.418	0.406	0.388	0.386
AES-MSH	0.468	0.454	0.415	0.415	0.431
BHIT-MSH	0.312	0.301	0.302	0.308	0.290

These coefficients are estimators based on the set of 500 simulations used to create Figs. 7 and 8

#### Results on WTCCC data

Performing genome-wide interaction association studies with several methods on the RA dataset remains a challenge, even after pruning the dataset as described in a previous section. Each of the methods discovered a large number of potential interactions when using the 5% threshold and the correction procedures described in the Methods section (ranging from 1805 for MSH to 3808 for MDR). In total, 1306 significant 2-SNP interactions were discovered by at least 2 methods: 12 by the 5 methods, 19 by 4 methods (see Table 4), 476 by 3 methods and 799 by 2 methods only (see Additional file 3 for a complete list). To obtain a ranked list of interactions, and although many sorting criteria could be used, we computed the rank of each interaction among the significant interactions of each method (the most significant interaction found using a given method was ranked 1 for that method, the second was ranked 2, etc. Interactions not present in the list of the given method were ranked (N + 1), where N is the number of significant interactions for that method). We then summed up the ranks obtained by each significant pair of SNP and sorted the list according to this sum (the smallest sum corresponding to the “best” interaction). The results for the 31 interactions detected by at least 4 methods are reported in Table 4.

**Table 4:**
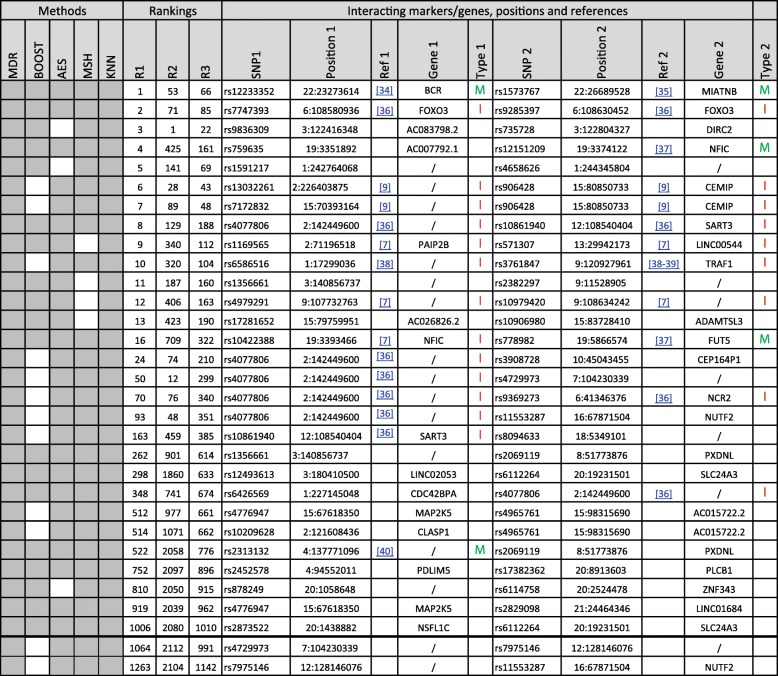
List of the significantly interacting SNP in the WTCCC RA data [7, 9, 34–40]

The 31 2-SNP interactions found significant by at least 4 methods. The corresponding SNP names and positions are also reported. Ref 1 and Ref 2 (when any) refer to previous studies where the corresponding SNP were already reported. Gene 1 and Gene 2 (when any) are reported when the corresponding SNP are located in a gene (in intronic, exonic or UTR regions). The methods for which the SNP were reported as significant are indicated by a colored cell. Furthermore, 3 rankings are also reported: the first one is the one used to rank the interactions in the Table and is described in the text. The second is the balanced accuracy computed by KNN-MDR. The third one is the rank of the average rank of the interaction computed over the methods for which this interaction was significant

In total, the 31 2-SNP interactions detected by at least 4 methods involve 47 distinct SNP (36 SNP are involved in only one interaction, 10 are involved twice and 1 is present in 6 interactions, see Table 4). Some interactions (12 out of 31) involve SNP on the same chromosome, while 19 involve SNP on distinct chromosomes. For intra-chromosomal interactions, the distance between the SNP ranged from very small (2 are smaller than 50 kb), to very large (2 are larger than 10 Mb). This shows that the methods potentially reported interactions involving close regions, such as upstream regulatory regions of genes, as well as much more distant ones, including regions located on different chromosomes. Several of these interactions have already been reported in previous analyses (see Table 4), while others are new, to our knowledge (for example on chromosome 3), or might potentially be echoes of other more significant ones.

**Fig. 11:**
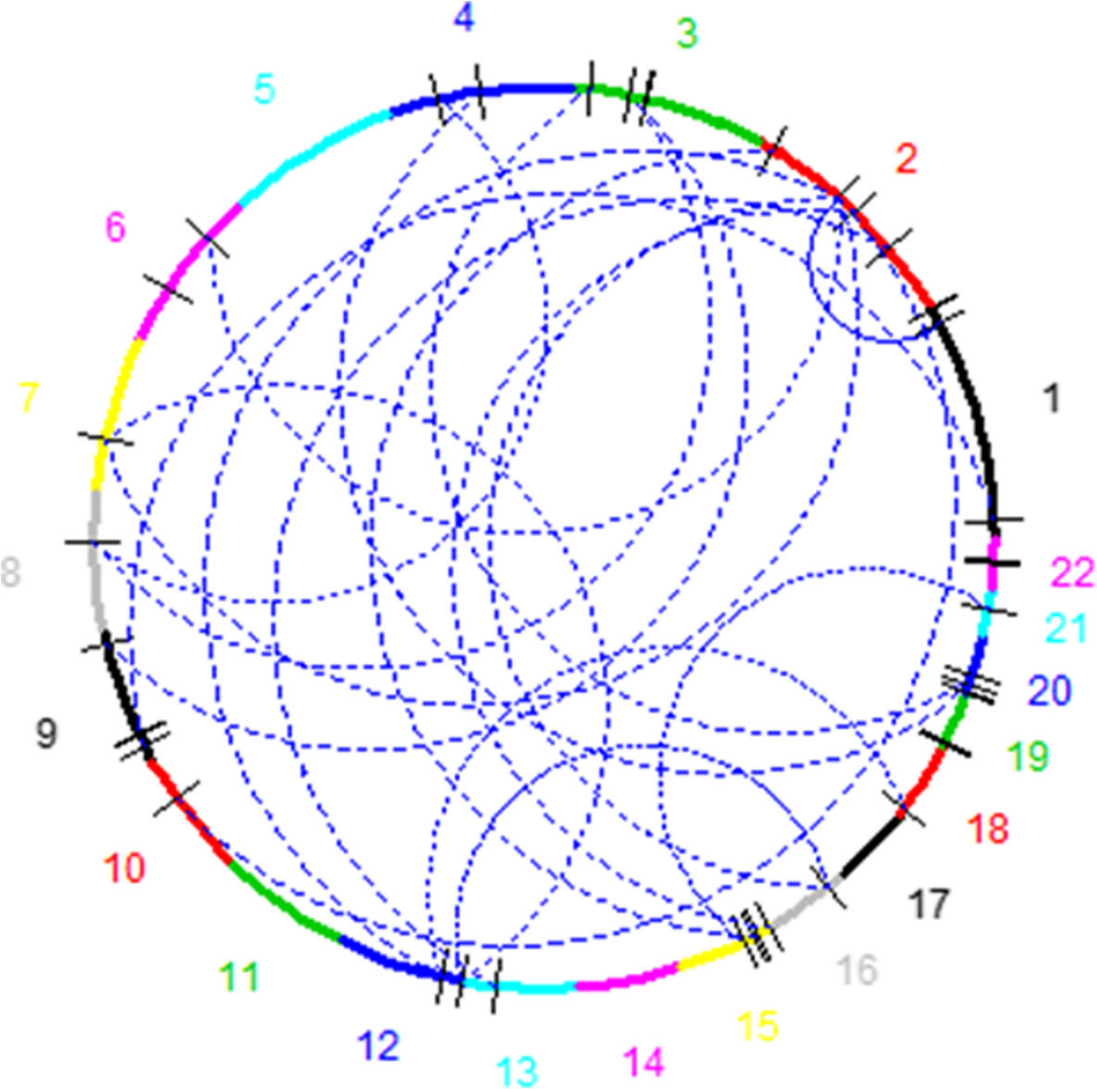
Results of the analysis on WTCCC data. Chromosomes showing significant interactions are reported in the graph. Interacting regions are denoted by ticks on the circle

Figure 11 provides another view of the results from this analysis (a Additional file 3 gives a more complete version of the results). On this figure, chromosomes are reported with a dimension approximatively proportional to their physical size, interacting sites are signaled through dashes corresponding to the location of the interacting SNP on the chromosome and the detected inter-chromosomal interactions are reported using the dashed lines within the circle.

## Discussion

The detection of genetic interactions is a notoriously difficult task, and, although numerous papers have been published in the field, a lot of work remains to be done to propose methodological advances allowing obtaining reliable and reproducible significant results in many gene-mapping studies. Our work aims to be a step in that direction.

A first difficulty is the statistical power issue to detect epistatic interactions: even if epistatic effects are not necessarily more tenuous than main effects, the number of tested hypotheses increases at least quadratically, making multiple testing corrections potentially more penalizing. Therefore, strategies allowing obtaining reasonable power in such studies are desirable. This is one of the features of the approach we propose in this paper. As shown in the Methods and the Results sections, aggregation strategies provide some potential increases in the detection power. Even if the power increases in the current study were rather modest, it has been shown that adding more methods in the aggregation should potentially increase the overall power. In our study, the theoretical expectations are supported by the simulation results (e.g. Fig. 5), with an improved power of the method aggregating the results of the 5 underlying methods with respect to the individual methods and to the methods aggregating less methods, although admittedly smaller than expected under the hypothesis of methods independence. Note that the property of independence mentioned here means that the probability of finding a positive result for one method does not depend on the findings of another method: although this might be arguable for ‘easy to find’ interactions, this might be more plausible for less ‘visible’ interactions, especially when distinct methods rest on very different approaches. Nevertheless, in our study, although we have used methods covering various methodologies (multi-dimensional reduction (MDR, KNN-MDR), exhaustive search (BOOST), empirical (AntEpiSeeker) and Bayesian (BHIT) approaches) and potentially hard to find interactions (small marginal effects, potentially heterogeneous situations, see the simulations description), we obtained significant correlations between the methods results (Table 3). Although the way these correlations affect the power is not completely clear, the global effect was a reduction of the obtained power increase compared to the expectation. This is also what was observed in a set of simulations involving correlated methods with known correlation structure (see Supplementary file 4).

Reproducibility is another problem in mapping studies. At least three reasons are at the root of this problem: a first reason is that many published results are probably false positives, partly due to improper correction for multiple testing. Another reason is that not every method is equally likely to detect any type of interaction, making detection not only a function of the variants to be highlighted, but also of the used method. And finally, and more fundamentally, it is to be expected that many phenotypes are under the control of many genes with intricate interaction networks. Consequently, involved interactions in one dataset, or even in subsets, might differ, increasing the heterogeneity of the underlying genetics and making detection of these interactions more complicated. Our approach is of interest for the two first problems. We have indeed shown that aggregating the positive results of various methods helps to control the false positive rates: false positives produced by one of the methods are not necessarily produced by the other used methods, and will most of the time be discarded from the final results (Figs. 7 and 8). On the other hand, positive results produced by a majority of methods will pop up, allowing combining the detection skills of several methods rather than only considering separately individual methods results. The definition of the majority might be important: although we have used a very simple approach to define the majority in our analyses, other schemes are possible. For example, we could weight each method differently in the majority calculation to better account for each method characteristics, with the weights inferred using regression techniques or simulations.

Aside of these interesting properties, some difficulties have to be mentioned. An obvious disadvantage of the aggregation strategy is that several methods have to be mastered, installed on the computer facilities and run. This of courses increases the total computing time, which might be an issue when large datasets are considered. A possible solution would be to use the nowadays largely available parallel resources offered in most research centers. Using several nodes to perform the tasks (run the programs implementing the various strategies, run the permutations when needed, etc.) in parallel should lead to a non-significant increase of the total observed run time, with a minor penalty due to the software implementation of this parallelization strategy. So, affordability of the aggregation strategy is a question about the affordability and applicability of the various individual methods, but not on the affordability and applicability of the aggregation strategy itself: extra efforts needed by the aggregation are really minor compared to the computational burden and needed user experience associated to the various methods.

Since more and more methods are becoming available in the context of ‘big data’, increasing the number of algorithms that can be tested and integrated in the aggregation should become more and more feasible in the coming years. Furthermore, improvements in the currently available methods are also possible. For example, Van Lishout et al. (2015) [41] analyzed ~ 10^6^ SNP on thousands individuals in 1 day on a 256 core cluster using MB-MDR! This was made possible by algorithmic improvements in the MB-MDR methodology and technical availability of large computers clusters. These evolutions should make aggregation a viable approach for large problems in the short term.

Another difficulty is the aggregation itself: the ranking of the interesting interactions is performed based on their significance. This leads to at least two problems: first, providing a clear ranking might be difficult; for example, when permutations are used, several interactions might easily end up with the same significance, making subsequent ranking almost arbitrary. Next, the most interesting combinations might not necessarily be the best-ranked ones even when an unambiguous ranking is available. Although this point clearly deserves more investigations, one possible crude approach, used in this study, was to incorporate more than the top-ranked combinations in the aggregation. Figures 9 and 10 show that this simple strategy has some merits, increasing the power while still controlling for the number of false positives when the number of kept top combinations increases from 1 to 5.

Our simulations modelled complex situations, with weak (or no) marginal effects and genetic heterogeneity. Such complex situations are not required to resort to aggregation, but our objective was to show the method performances in settings where individual are not able to systematically discover the simulated interactions.

In view of the main characteristics of our strategy, it was important to test the approach on real datasets to check whether new clues could result from our analyses. Our results on the WTCCC Rheumatoid Arthritis data provides new information on potential new candidate regions. As shown in Table 4, we rediscovered several previously reported associations and interactions in our study. Furthermore, interactions between previously identified genes and other new genes or regulatory regions have also been highlighted, which can possibly provide new and useful information on the molecular mechanisms leading to RA. Finally, entirely new interactions are also found significant in our study, which might point to new target genes to be investigated in future RA studies, although their biological relevance is obviously not clear at this stage.

Figure 11 provides a view of the significant results at the chromosome level. This Figure and Table 4 show that some interactions involve SNP on 2 distinct chromosomes while other involve (sometimes closely) linked SNP. Although this might make biological sense (for example, regulatory regions might be close to the genes they influence; alternatively, very close markers could actually define haplotypes that are associated to the trait, a situation which is a special case of interactions that could probably be better flagged using haplotype-based methods), a potential bias of our method has to be mentioned. Indeed, BOOST tends to detect much more internal interactions than interactions between different chromosomes segments [11]. In our analyses, we also noted that less interactions involving closely linked SNP were detected when BOOST was removed from the aggregation (data not shown). Consequently, adding other (maybe less biased?) methods and/or somehow relaxing the majority vote criterion might allow to uncover (and maybe also exclude) other combinations.

## Conclusions

In summary, the aggregation of methods is an approach with interesting features for detecting epistatic interactions. Integrating the results of parallel methods should increase the corrected power, making aggregation more powerful than the individual methods while controlling the false positives rate. We also have demonstrated the feasibility of using such methodology on real genome-wide datasets, providing potential new insights in complex traits analyses.

## Additional files

Additional file 1: Some simulations results using a known correlation structure between the individual methods. (DOCX 25 kb)

Additional file 2: The results of 6 individual methods and the aggregation method on the 1000 simulations by taking the first significant combination. (XLSX 330 kb)

Additional file 3: List of the significantly interacting SNP in the WTCCC RA data for the results found by every method. (XLSX 164 kb)

Additional file 4: The results of 6 individual methods and the aggregation method on the 500 simulations by taking the first 5 significant combinations. (XLSX 177 kb)

## Abbreviations

BHIT: Bayesian High-order Interaction Toolkit
BOOST: Boolean operation-based screening
FAM-MDR: Flexible family-based multifactor dimensionality reduction
GMDR: Generalized multifactor dimensionality reduction
GWAS: Genome-wide association study
HSA9: Human chromosome 9
KNN: K-nearest neighbors
LD: Linkage disequilibrium
MAF: Minor allele frequency
MB-MDR: Model-based multifactor dimensionality reduction
MDR: Multi dimensional reduction
SNP: Single-nucleotide polymorphism
WTCCC: Wellcome trust case control consortium
