# Supplementary material for: Aggregation of experts: an application in the field of “interactomics” (detection of interactions on the basis of genomic data)
Source: BMC Bioinformatics. 2018 Nov 21;19:445. doi: 10.1186/s12859-018-2447-0 (PMC6267805; doi:10.1186/s12859-018-2447-0)
Supplement: Supplementary file 1 — Some simulations results using a known correlation structure between the individual methods. (DOCX 25 kb) [file 12859_2018_2447_MOESM1_ESM.docx]

# Additional file 1

In order to evaluate the effect of the correlation between methods on the aggregated predictor, we have performed the following simulations:

- Similarly to Table 1, we have assumed that all the individual methods had equal power. This is no restriction, all combinations of powers could be tested similarly.
- We have assumed that the aggregator used N = 10 methods, as an example.
- In order to cover a large range for the powers Π of the individual methods, we have used individual powers of 0.2, 0.3, 0.4, 0.5, 0.6, 0.7 and 0.8.
- The correlations ρ between the methods (see below) have been set to values from 0.0 to 0.9 by steps of 0.1.
- For each tested correlation, a threshold T was computed as $T= \Phi^{-1}\left( \Pi\right)$ where $\Phi$ is the standard normal distribution. Consequently, if x has a standard normal distribution, $P\left( x<T \right)=\Pi$.
- For each combination (Π, ρ), we have performed 5000 simulations as follows:
  - We assumed that a vector **y** of N values were randomly sampled from a multivariate normal distribution N(**µ**, **Σ**), where **µ** = **0** is a null vector of size N and **Σ** is the variance covariance matrix, set to:

$$\boldsymbol{\Sigma}= \left( \begin{matrix} 1 & \begin{matrix} \rho& \cdots& \rho\end{matrix} \\ \begin{matrix} \rho\\ \vdots\\ \rho\end{matrix} & \begin{matrix} 1 & \cdots& \rho\\ \vdots& \ddots& \vdots\\ \rho& \cdots& \rho\end{matrix} \end{matrix} \right)$$

  - The values below T in **y** were considered as detections for the corresponding methods.
  - When several (K) methods “detected” something, we considered that the aggregated method also detected. The value of K could be 2 (in situations where 2 methods detect the same region, while the (N-2) remaining methods either do not detect a region, or detect different regions. So, the majority vote would choose the region selected by the 2 first methods) or more. The figures below show the results for K = 3 and K = 4 (it is unlikely than more than K = 4 would be needed to obtain a majority vote).
- Results

| 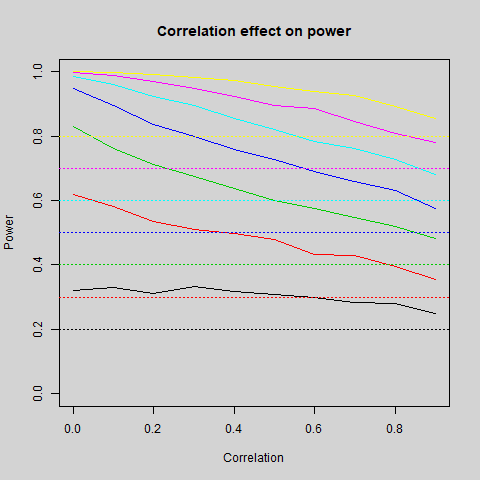 | 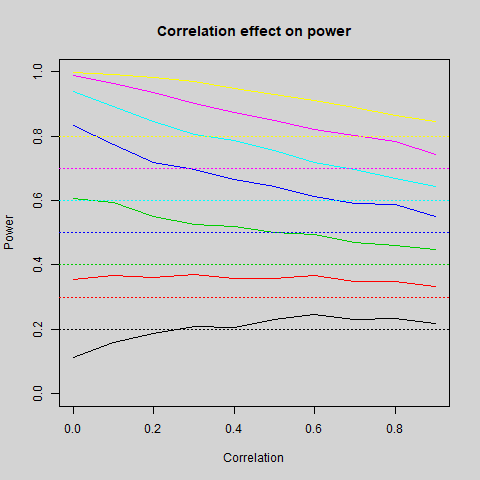 |
| --- | --- |
| **Figure 4.1** : simulation results for the power of the aggregated method when K = 3 (left graph) or K = 4 (right graph) are considered to declare a « majority ». The solid line are the power curves for the aggregated method while the dotted lines provide the individual methods power. The colors denote the individual methods power, from 0.2 to 0.8. | |

The results show that:

- In most cases, the power decreases when the correlation between the methods increases.
- In almost all cases, the aggregated method outperforms the individual methods in terms of power.
